# Understanding the mechanisms of resistance to azole antifungals in *Candida* species

**DOI:** 10.1093/jacamr/dlaf106

**Published:** 2025-06-23

**Authors:** Yunxiao Li, Charlotte Hind, Jessica Furner-Pardoe, J Mark Sutton, Khondaker Miraz Rahman

**Affiliations:** School of Cancer and Pharmaceutical Science, King’s College London, London SE1 9NH, UK; Antimicrobial Discovery Development and Diagnostics Team, UK Health Security Agency, Porton Down SP4 0JG, UK; Klura Labs, The Epicentre, Haverhill Research Park, Enterprise Wy, Haverhill CB9 7LR, UK; School of Cancer and Pharmaceutical Science, King’s College London, London SE1 9NH, UK; Antimicrobial Discovery Development and Diagnostics Team, UK Health Security Agency, Porton Down SP4 0JG, UK; School of Cancer and Pharmaceutical Science, King’s College London, London SE1 9NH, UK

## Abstract

Cases of *Candida* infection have been on the rise in recent years. A comprehensive and clear understanding of the mechanisms of antifungal resistance is fundamental for developing novel therapies to address the current and emerging threat of fungal diseases. Certain *Candida* species can cause superficial or invasive infections in immunocompromised hosts, and invasive *Candida* infections are major contributors to infectious disease deaths. As fungi are eukaryotes like humans, there are only a limited number of unique molecular targets available for antifungal drug development. Until recently, there have only been four primary classes of antifungals used to treat systemic fungal infections. Among these, azole antifungals are globally used because they are both inexpensive and effective. Due to various factors, resistance to antifungal drugs—especially azole antifungals—has developed in many *Candida* species, posing a significant public health threat. This review discusses the known mechanisms of azole antifungal resistance in *Candida albicans*, *Candida auris*, *Nakaseomyces glabrata*, *Candida tropicalis*, *Candida parapsilosis* and explores strategies to overcome the resistance problem.

## Introduction

Fungal infections, ranging from superficial to invasive, affect the lives of more than a billion people worldwide and contribute to more than 1 million deaths every year.^1,2^ Invasive candidiasis (IC) includes a range of clinical conditions, with candidemia being the most common. It is associated with a high mortality rate, particularly among critically ill and immunocompromised patients.^3^ The overall mortality rate for IC is reported to be over 30%, despite available treatments. In cases where patients present with septic shock, the mortality rate can exceed 50%.^4^ Additionally, candidemia is associated with an in-hospital all-cause mortality of approximately 25%.^5^ The economic cost of *Candida* infections, particularly IC, imposes a substantial financial burden on the healthcare system. High-income countries face significant direct medical expenses. The mean total cost per patient with *Candida* infections was estimated to be more than $50 000 in direct healthcare costs in the United States in 2017.^6^ Low- and middle-income countries likely face higher morbidity and mortality due to limited resources and access to advanced treatments. The number of cases of IC is increasing dramatically due to many factors, including a growing number of patients with weakened immune systems, such as those with HIV, cancer and chronic diseases of the respiratory tract, kidneys, or liver, as well as interventions such as normal cancer therapy and organ transplantation.^7,8^ In the last 4 years, the COVID-19 pandemic has been reported to be associated with an increase in the incidence of IC due to several interrelated factors, including (i) that severe COVID-19 can lead to a compromised immune system, (ii) patients with severe COVID-19 often require long-term hospitalization and ventilator assistance, which increases their risk of hospital-acquired infections and (iii) COVID-19 patients often receive broad-spectrum antibiotics, which disrupt the normal microbiota and allow opportunistic fungi like *Candida* to overgrow.^9–13^

**Figure 1.**
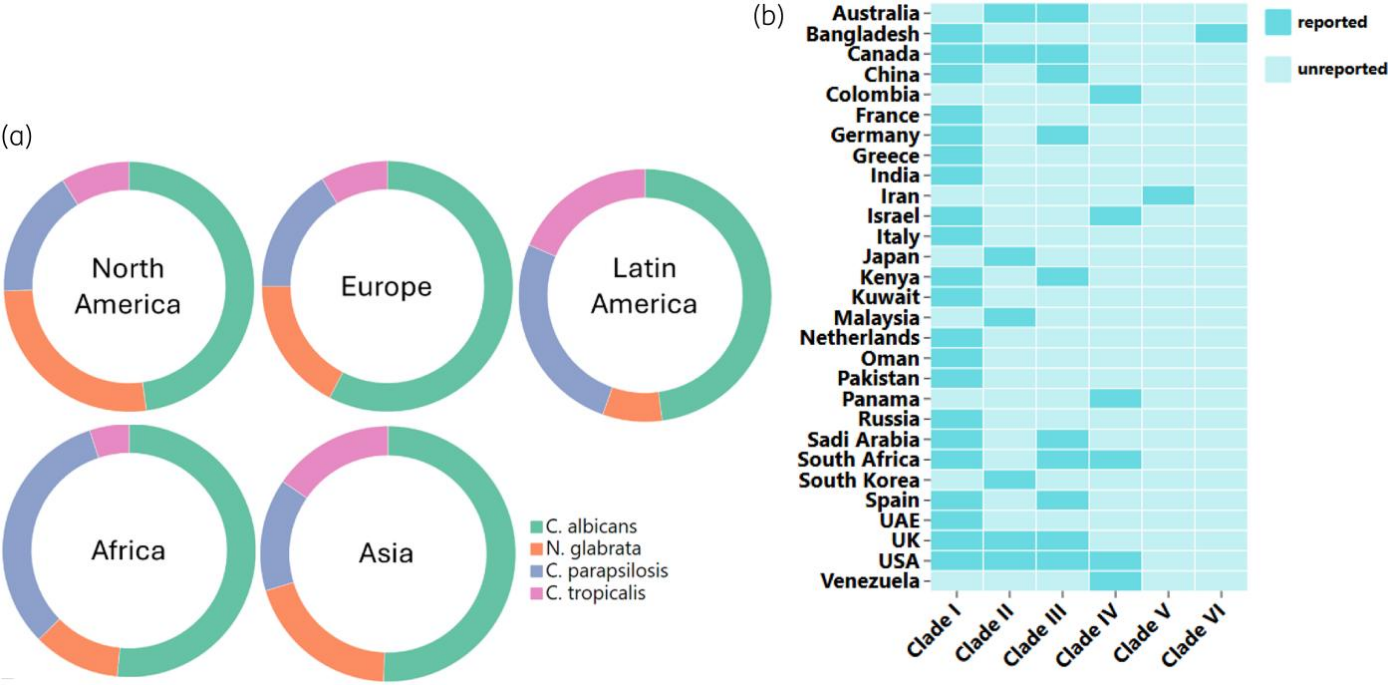
a) Species distribution of *Candida* species. b) Six clades of *Candida auris* reported from 17 countries (drawn using Excel and Chiplot).

*Candida* species are recognized as one of the fungal pathogen groups responsible for the majority of severe fungal infections. In 2022, the WHO Fungal Priority Pathogens List (FPPL) to guide research, drug development and public health interventions, ranked *Candida auris* and *Candida albicans* in the ‘critical’ priority group, and *Nakaseomyces glabrata* (previously *Candida glabrata*), *Candida tropicalis* and *Candida parapsilosis* in the ‘high’ priority group.^8^ These species vary in prevalence and distribution due to different climates, healthcare practices, antimicrobial use patterns and local epidemiological trends. Although a decrease in the proportion of *C. albicans* infections has reduced over time compared with non-albicans *Candida* species, *C. albicans* remains the leading cause of IC.^5,14^ For non-albicans species, *N. glabrata* and *C. parapsilosis* are common in North America and Europe, *C. parapsilosis* and *C. tropicalis* are common in Latin America, *C. tropicalis* and *N. glabrata* are common in Asia, and *C. parapsilosis* is the most common in Africa (Figure 1a).^15–17^

*C. auris* was first isolated in 2009 from the external ear canal of a patient in Japan.^18^ Six genetically distinct *C. auris* clades have shown geographical characteristics: Clade I (South Asian), Clade II (East Asian), Clade III (South African), Clade IV (South American), Clade V (Iran) and Clade VI (Bangladesh).^19,20^ The prevalence of *C. auris* globally is not well tracked, and only 29 countries have recorded different clades causing *Candida* diseases (Figure 1b).^19,21–23^ Due to its properties of nosocomial transmission, causing life-threatening infections, and multidrug resistance, *C. auris* should be a reportable species global.^24^

Only three main classes have been available for the systemic treatment of IC: the polyenes (Amphotericin B, Nystatin), the azoles (Fluconazole, Itraconazole, Voriconazole) and the echinocandins (Caspofungin, Micafungin, Anidulafungin).^25–28^ The azoles are the most commonly used antifungal drug class in clinical settings due to their broad spectrum of activity, good tolerability and good tissue penetration.^29^ New classes of antifungal agent have recently been licenced, including for the treatment of *Candida* infections.

After 1958, when the first azole antifungal, chlormidazole, was introduced to the market, four generations of azoles, including about 40 azole-containing drugs and candidates, were developed over the next 50 years.^30,31^ All of them can be classified into three groups depending on the structure of their rings: imidazoles (ketoconazole, miconazole and econazole) have two nitrogens in the azole ring, triazoles (e.g. fluconazole, itraconazole, posaconazole, isavuconazole and voriconazole) have three nitrogens in the azole ring, and tetrazoles (e.g. oteseconazole, quilseconazole) have four nitrogens in the azole ring. In the clinic, treatment modalities for *Candida* infections can be divided into topical and systemic treatments. The use of imidazoles, with the exception of ketoconazole, is limited to topical treatments, whereas triazoles, being less toxic and more specific than imidazoles, are suitable for systemic treatments.^32^ In the clinic, three triazoles are recommended for the systemic treatment of IC: fluconazole, miconazole and clotrimazole.^28,33–35^ It should be noted that the use of azoles in agriculture representing at least 25% of all fungicides sprayed on crops has increased significantly since the mid 2000s and although the specific drugs differ (e.g. prothioconazole, epoxiconazole and tebuconazole), they share the same mechanisms of action and may play a role in selection for resistance in human pathogens.^36^ Azoles such as ketoconazole and climbazole are also found in a number of consumer products, such as toothpaste.^37^ This is one of the few examples where drugs, which are critically important for the treatment of life-threatening infections in people have analogues with such widespread usage in the environment, and the impact of this on drug resistance, in both sectors has been highlighted as a concern.^38^

**Figure 2.**
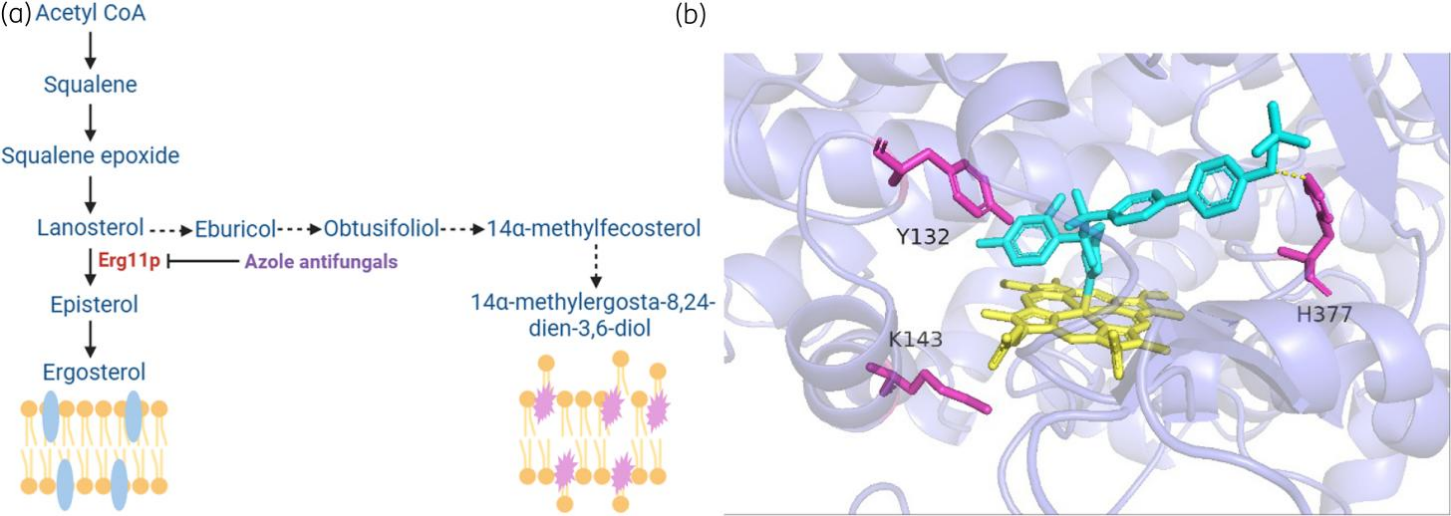
a) The scheme of ergosterol biosynthesis. Erg11p is an enzyme that catalyzes the conversion of lanosterol to episterol. Lanosterol contains a C14 methyl group, which is oxidatively removed by the cytochrome P450 enzyme Erg11p. Azole antifungals inhibit Erg11p, thereby blocking the demethylation step critical for ergosterol biosynthesis. When it is inhibited by azole antifungals, the toxic sterol 14α-methylergosta-8,24-dien-3,6-diol accumulates in the cell. b) The interaction between oteseconazole, which was approved by FDA in April 2022 and is one of the strongest antifungals (in blue) and iron (in yellow) in Erg11p of *Candida albicans*.^39^ Y132 and K143 (in pink) are key sites for mutations leading to azole resistance, and H377 can form a hydrogen bond with tetrazole.

Azole antifungals can inhibit the synthesis of ergosterol by targeting the cytochrome P450 enzyme sterol 14α-demethylase, or Erg11p, which catalyzes the oxidative removal of the sterol C-14 methyl group (Figure 2a).^40^ When azole antifungals bind to Erg11p, the azole ring coordinates with the iron, which is essential for the enzyme's demethylation of lanosterol (Figure 2b).^41,42^ Ergosterol is a crucial sterol in the fungal cell membrane, involved in maintaining cell structure and functions such as enzyme activity and nutrient transport.^43^ Inhibition of Erg11p leads to the accumulation of sterol precursors, resulting in alterations to the structure and function of the cell membrane.^44^ The accumulation of hazardous metabolites, which also results from the inhibition of the ergosterol synthesis pathway, such as 14α-methylergosta-8,24-dien-3,6-diol produced from 14-methylfecosterol, has been reported to be responsible for growth arrest.^45^

Although azole antifungals are heavily relied on, having played an important role in the treatment of both systemic and topical *Candida* infections, many azole antifungals have been rendered less effective by the rapid emergence of antifungal resistance.^46–50^  *Candida albicans*, *N. glabrata* and *C. parapsilosis* exhibit higher resistance to itraconazole and fluconazole compared to other azole antifungals.^35,51–54^ Among all reported azole antifungals, fluconazole has the highest incidence of drug resistance, with 93% of *C. auris* reported to be resistant to fluconazole.^55^ Clotrimazole resistance, which used to be rare, is now quite common in certain patients with candidiasis and may be associated with cross-resistance to other azole antifungals.^56^ The rates of azole-antifungal resistance in various *Candida* species differ and may depend on factors such as different species and geographical location (Table 1). *Candida albicans* generally exhibits the lowest resistance rates, whereas non-albicans species such as *N. glabrata* and *C. tropicalis* exhibit higher resistance rates in certain regions.^64,65^ Of particular concern is the emergence of *C. auris* and resistant clones of *C. parapsilosis*, which can cause difficult-to-control outbreaks in hospital settings.^66–68^ This review will focus on the current resistance phenomenon and mechanisms of azole antifungals used in the treatment of invasive *Candida* infections and related species.

**Table 1.** The rate of azole antifungal resistance in different Candida species and the areas where resistance has been reported (1994–2024)

Species	Typical azole resistance rates	Notable characteristics and geography
*Candida. albicans* ^14^	Low (≤5% in most settings); Mostly susceptible to all azoles.	Increased resistant strains were reported by a few hospitals but in general, no major geographic hot-spot. Intrinsic susceptibilities: fully susceptible to azoles unless resistance acquired.
*Nakaseomyces glabrata* ^14,57^	Moderate and rising (10%–20% fluconazole-resistant in U.S.; about 5%–10% in Europe; lower in LATAM/Asia)	Regional: Highest in North America; also a concern in Europe. Frequently cross-resistant to all azoles once resistant.
*C. auris* ^55,58,59^	Very high (>90% fluconazole-resistant globally; voriconazole resistance 3%–73% depending on clade)	Global spread: South Asia, Americas, Africa, etc. Multidrug-resistant, azoles largely ineffective (especially fluconazole). Persists on surfaces, causes outbreaks in hospitals. Some clade variation (e.g. East Asian clade less azole-resistant)
*C. tropicalis* ^14,60,61^	Increasing (Asia-Pacific up to 20%–30% fluconazole-resistant; other regions still low).	Regional: Problematic in Asia (India, China, Southeast Asia); much lower in US and EU. Environmental reservoirs may contribute.
*C. parapsilosis* ^14,62,63^	Generally low (<5%), but outbreak-associated increase (up to 50%–80% in hospital clusters)	Regional: South Africa had high background resistance; outbreaks also in South America, Europe, Asia. Hospital surfaces and hand transmission are key in spread.

## Mechanism of azole antifungal resistance

Fungal strains are classified as non-susceptible to azole antifungals when their MIC is higher than the breakpoints defined by CLSI/EUCAST, encompassing dose-dependent susceptible, intermediate susceptibility (SDD or I—Intermediate, respectively) and resistant (R) isolates (Table 2).

**Table 2.** Clinical breakpoints for azole antifungals in *Candida* species (in mg/L)

	Fluconazole	Itraconazole	Voriconazole	Posaconazole	Isavuconazole
*Candida Albicans*	CLSI: S ≤ 2, SSD =4, R ≥ 8; EUCAST: S ≤ 2, R > 4	CLSI: IE; EUCAST: S ≤ 0.06, R > 0.06	CLSI: S ≤ 0.12, SSD = 0.25–0.5, R ≥ 1; EUCAST: S ≤ 0.06, R > 0.25	CLSI: IE; EUCAST: S ≤ 0.06, R > 0.06	CLSI: IE; EUCAST: IE
*Nakaseomyces glabrata*	CLSI: SDD ≤32, R ≥ 64; EUCAST: S ≤ 0.001, R > 16	CLSI: IE; EUCAST: IE	CLSI: IE; EUCAST: IE	CLSI: IE; EUCAST: IE	CLSI: IE; EUCAST: IE
*C. parapsilosis*	CLSI: S ≤ 2, SSD =4, R ≥ 8; EUCAST: S ≤ 2, R > 4	CLSI: IE; EUCAST: S ≤ 0.125, R > 0.125	CLSI: S ≤ 0.12, SSD = 0.25–0.5, R ≥ 1; EUCAST: S ≤ 0.125, R > 0.25	CLSI: IE; EUCAST: S ≤ 0.06, R > 0.06	CLSI: IE; EUCAST: IE
*C. tropicalis*	CLSI: S ≤ 2, SSD =4, R ≥ 8; EUCAST: S ≤ 2, R > 4	CLSI: IE; EUCAST: S ≤ 0.125, R > 0.125	CLSI: S ≤ 0.12, SSD = 0.25–0.5, R ≥ 1; EUCAST: S ≤ 0.125, R > 0.25	CLSI: IE; EUCAST: S ≤ 0.06, R > 0.06	CLSI: IE; EUCAST: IE

CLSI/EUCAST has not established specific breakpoints for azole antifungals in *C. auris*. ‘S’ stands for susceptible, ‘SDD’ for susceptible dose-dependent, ‘R’ for resistant, and ‘IE’ for insufficient evidence. Information taken from CLSI M27M44S, Performance Standards for Antifungal Susceptibility Testing of Yeasts, 3rd Edition; EUCAST Clinical breakpoints for fungi v. 11.0 valid from 2 December 2024; accessed 1 May 2025.

**Figure 3.**
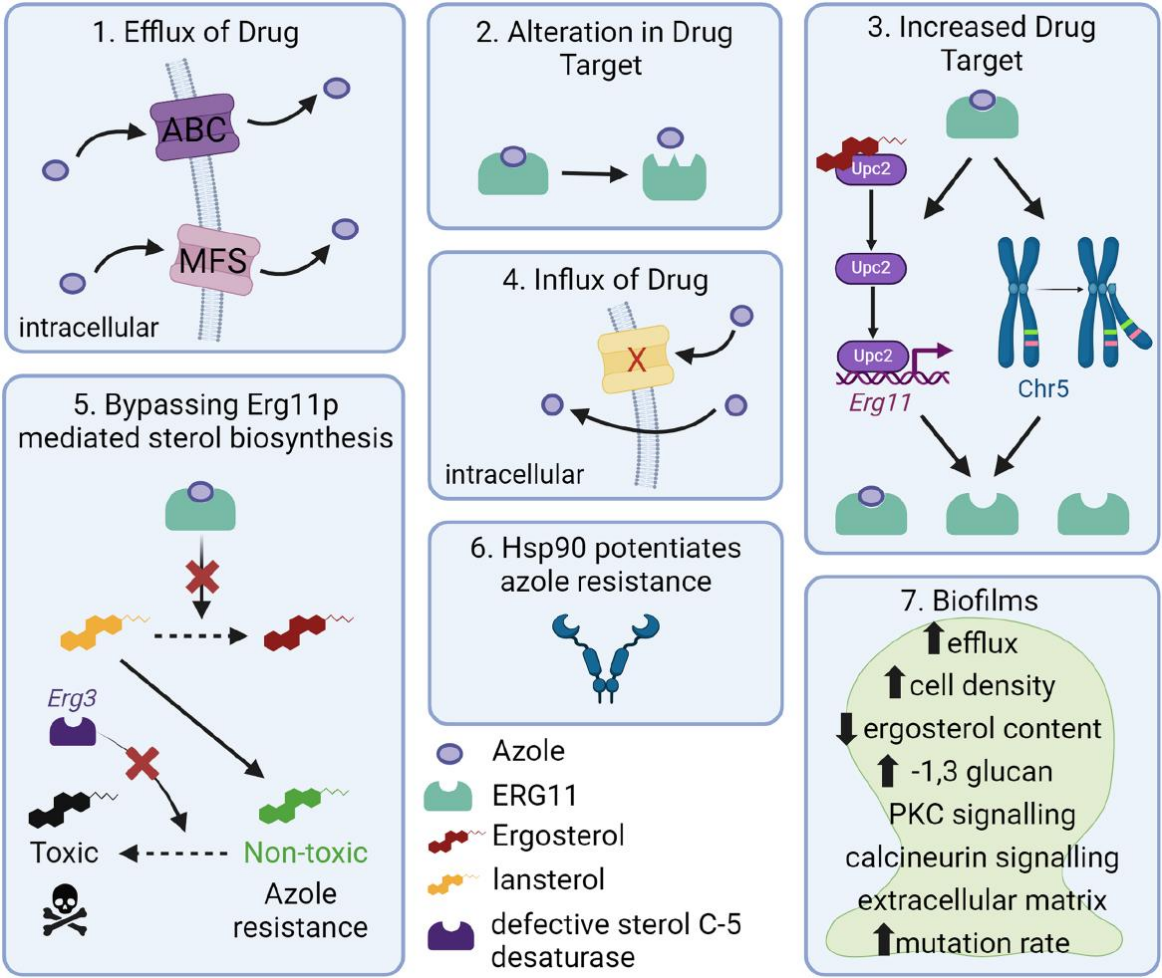
Mechanisms of azole antifungal resistance in *Candida* species. 1. Azoles are effluxed through upregulated ABC and MFS transporters; 2. Mutations in *ERG11* cause alterations in the drug target, Erg11p; 3. Increased expression of the drug target through the release of the transcription factor Upc2 from ergosterol, or aneuploidy of chromosome 5 which contains *ERG11* and the transcription factor *TAC1,* also resulting in upregulated efflux; 4. Reduced influx of azoles through transporters; 5. Inhibition of the Erg11p mediated sterol biosynthesis of lanosterol to ergosterol caused by azoles would usually result in a toxic sterol byproduct, 14α-methylergosta-8,24-dien-3,6-diol, unless loss-of-function mutations arise in *ERG3,* whereby Erg3p does not convert the non-toxic sterol byproduct, 14α-methylsterol, to the toxic one, and azole resistance emerges; 6. Stress response pathways such as Hsp90 can potentiate azole resistance; 7. Multiple resistance mechanisms within biofilms result in azole resistance. Created using BioRender software.

There are multiple mechanisms which can cause azole antifungal resistance in *Candida* species. Major molecular mechanisms associated with azole resistance in *Candida* species include (i) increased expression of the drug target, including through increased genome copy, (ii) alteration in the drug target, (iii) increased drug efflux and (iv) alterations in sterol biosynthesis (Figure 3). Of all the different mechanisms, one of the most studied genes for understanding azole antifungal resistance is *ERG11*, which encodes the Erg11p targeted by azoles, while overexpression of genes encoding efflux pumps is considered the most common mechanism.^69,70^

### Increased drug efflux

Overexpression of efflux pumps has been identified as the major mechanism in clinical high-level azole resistance in *Candida* isolates, especially for *C. albicans*.^71,72^ The ATP-binding cassette (ABC) and the major facilitator superfamily (MFS) transporters are the main families associated with azole resistance in these isolates.^72^  *Candida* cells contain a range of ABC and MFS proteins, but only a few have a clear role in clinical drug resistance (Table 3). Cdr1p and Cdr2p in the ABC family, and Mdr1p in the MFS family, stand out for their clinical relevance.^73–75^

**Table 3.** Summary of mechanisms of azole antifungal resistance in Candida species

Mechanism	Gene(s) involved	Regulator(s) involved
Efflux pump overexpression		
ABC transporters	*CDR1*, *CDR2*, *CDR3*, *PDH1*, *SNQ2*	*TAC1*, *GPX1*, *RTA3*, *EBP1*, *PDR1*
MFS transporters	*MDR1*, *FLU1*, *MDR2*	*CPH1*, *MRR1*, *UPC2*, *MCM1*
Drug target		
Drug target alteration	*ERG11*	
Drug target overexpression	*ERG11*	*UPC2*
Aneuploidy	*ERG11, TAC1*	
Bypass sterol biosynthesis	*ERG3*	*EFG1*

ABC, ATP-binding cassette; TMF, major facilitator superfamily.

#### ABC Transporters

ABC transporters can be found in all organisms and are among the largest and most highly conserved known protein superfamilies and act as drug efflux pumps in *Candida* species.^76,77^ Several putative ABC proteins were identified and classified into six distinct subfamilies in the genome of *C. albicans*, including the pleiotropic drug resistance (PDR) the multidrug resistance (MDR), the multidrug resistance-associated protein (MRP), the adrenoleukodystrophy protein (ALDp), the elongation factor-3 (EF3) and the RNase L inhibitor (RLI), but only CaCdr1p and CaCdr2p which belongs to PDR subfamily have been demonstrated to be highly associated with azole resistance.^73–75^

**Figure 4.**
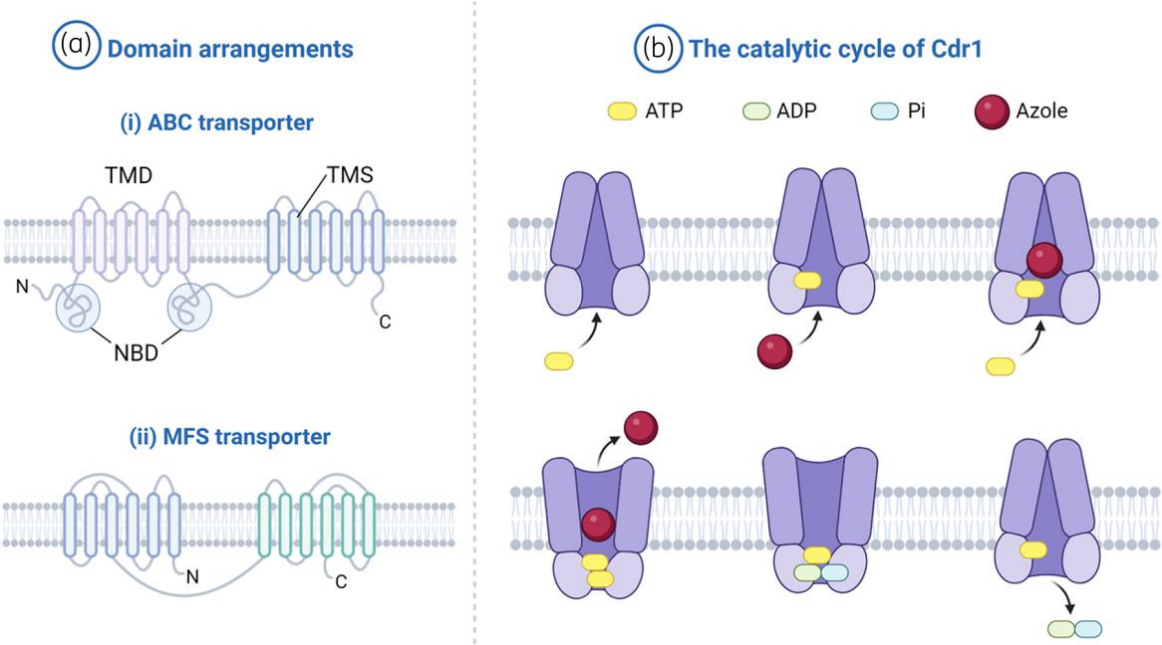
a) (i) ABC and (ii) MFS transporters of *Candida* species. The topology shown here features the (NBD-TMS_6_)_2_ arrangement for ABC transporters and the (TMS)_12_ arrangement for MFS transporters. b) The catalytic cycle of the ABC transporter Cdr1. NBD,  nucleotide binding domain; TMD,  transmembrane domain; TMS,  transmembrane segment. Created using BioRender software.

Cdr1p and Cdr2p consist of two transmembrane domains (TMDs) and two nucleotide-binding domains (NBDs). Each TMD spans the membrane six times via putative α-helices transmembrane segments (TMSs), and the NBDs are the sites for ATP binding and hydrolysis (Figure 4a).^72,78,79^ Conformational changes in transporters, triggered by ATP binding and hydrolysis, can lead to the pumping out of azole drugs. At least one substrate-binding site in each half of the transporter has been identified for both Cdr1p and Cdr2p, but only the mechanism of action for Cdr1p has been clearly reported.^80^ Initially, in the inward-open conformation of Cdr1p, one ATP molecule may bind to NBD1 without hydrolysis, while a second ATP molecule binds to the catalytically active NBD2. This interaction causes a rigid body motion that switches the transporter to the outward-open conformation, providing a pathway for substrates, including azole drugs, to exit the transporter (Figure 4b).^81^

Overexpression of both *CDR1* and *CDR2*, which encode the Cdr1p and Cdr2p proteins, has been found to be synergistic in reducing the susceptibility of *C. albicans* to azole antifungals.^82–84^ A *C. albicans* strain with both CaCdr1 and CaCdr2 deleted, resulting in the double cdr1Δ:cdr2Δ mutant, was more susceptible to azoles than a strain with a single cdr1Δ mutant.^74^ Interestingly, evidence indicates that CaCdr1 may be more critical than CaCdr2 in fluconazole-resistant *C. albicans* clinical isolates,^72,80,84^ but other claimed that the overexpression of *CDR2* plays an equal or even greater role than *CDR1* in the azole resistance of *C. albicans* because the *CDR2* expression reverted cdr1Δ mutant isolates to the wild-type level of susceptibility to azoles.^74^

In *N. glabrata*, three genes coding for ABC transporters, *CgCDR1*, *CgCDR2* (*PDH1*) and a distinct ABC transporter gene *CgSNQ2*, have been identified. The proteins encoded by *CgCDR1* and *CgCDR2* share 73% amino acid sequence identity.^85,86^ Although expression levels of both *CgCDR1* and *CgCDR2* were increased in azole-resistant *N. glabrata* isolates, only the function of *CgCDR1* as a multidrug transporter has been demonstrated.^85,87,88^ Interestingly, disruption of *CgPDR1* (the transcriptional regulator gene) can eliminate expression of *CgSNQ2* gene, and the P822L mutation in *CgCDR1* results in *CgSNQ2*-mediated azole resistance.^86^ In *C. parapsilosis*, the expression of the *CDR1* ortholog of *C. albicans* does not influence azole susceptibility, and the *CDR2* ortholog was not identified.^89^ In *C. tropicalis*, in addition to *CDR1* and *CDR2*, the overexpression of a distinct pump *CDR3* was also reported in azole-resistant strains.^90^

The overexpression of efflux pumps is correlated with many factors. Gain-of-function (GOF) in Tac1p (a zinc-cluster transcription factor activating *CDR1* and *CDR2*) represent a known mechanism in *C. albicans* isolates. Missense mutations in *TAC1* (switching heterozygosity to homozygosity) leading to hyperactive alleles is associated with a constitutively high expression of *CDR1* and *CDR2* and can upregulate *CDR1* and *CDR2* genes.^72,91,92^ Additionally, the upregulation of two coordinately regulated genes, the glutathione peroxidase gene *GPX1* and the drug resistance gene *RTA3*, as well as the downregulation of the drug resistance gene *EBP1*, have been identified in isolates with upregulated *CDR1* and *CDR2*.^93^ Similar to *C. albicans*, mutations in *TAC1B*, which encodes a transcription factor highly homologous to CaTac1p, have been associated with significantly increased expression of *CDR1* in azole-resistant *C. auris*.^94^

*Candida* species, especially the diploid *C. albicans*, exhibit notable genomic plasticity, and aneuploidy can contribute to drug resistance. Under azole pressure, *C. albicans* and *C. auris* can duplicate or amplify portions of its genome that carry resistance genes. The formation of an isochromosome of the left arm of chromosome 5 (i5(L)), frequently observed in azole-resistant *C. albicans* isolates, causes 2-fold amplification of dozens of genes, including *ERG11* (azole target) and *TAC1* (transcriptional activator of CDR pumps), leading to simultaneous overexpression of the drug target and efflux pumps.^95–97^ This dual gene dosage increase confers high-level fluconazole resistance and represents one of the first described aneuploidy-mediated antifungal resistance mechanisms.

In *N. glabrata*, mutations in *CgPDR1* (a transcription regulator gene) have been confirmed to be associated with increased susceptibility to azole antifungals, and the increased expression of *CgCDR1* and *CgCDR2* is controlled by the overexpression of *CgPDR1*.^98,99^ These mutations typically occur in specific regions of CgPdr1 (e.g. inhibitory or transcriptional activation domain) and result in constitutive pump activation.^100,101^ Consequently, azole-resistant *N. glabrata* isolates often show cross-resistance to *all* azoles due to broad-spectrum efflux.^102^ In addition, despite being haploid, *N. glabrata* can undergo chromosomal alterations including segmental duplications and the formation of new minichromosomes to amplify genes associated with resistance.^103^

#### MFS Transporters

The MFS consists of secondary carriers that transfer substances by responding to chemiosmotic ion gradients. In the *C. albicans* genome, different MFS proteins have been clustered into 17 families, with each family typically transporting a specific type of compound.^104^ They consist of three different types of porters: symporters, antiporters, and uniporters, and the transporter spans the membrane either 12 or 14 times.^105^ Two families are associated with multiple drug resistance, including azole resistance: DHA1 (Drug H + Antiporter 1, 12 spans) and DHA2 (Drug H + Antiporter 2, 14 spans).^104,106^ On average, DHA1 and DHA2 have more genetic variation than ABC transporters, and DHA2 is more variable between transporters in different species than DHA1.^106^

Only a few DHA transporters are associated with azole antifungal drug resistance in fungi. In azole-resistant *C. albicans* isolates, only two MFS transporters, Mdr1p and Flu1p, belonging to DHA1, have been observed.^107^ CaMdr1p has been identified as a transporter for fluconazole (in some reports, ketoconazole and voriconazole also but specificity for other azoles remains untested), but the disruption of *Flu1p* encoding gene has little effect on fluconazole susceptibility.^72,108–110^ The different substrate recognition may be influenced by molecular size and hydrophobicity of the azole compounds. Fluconazole, being smaller and less hydrophobic, is more easily recognized and expelled by Mdr1p.^111,112^ The structure of Mdr1p in *C. albicans* has been characterized, consisting of 564 amino acids (∼63 kDa) and 12 TMSs (Figure 4a).^113^ Similar to ABC transporters, all TMSs are divided into two TMDs and are interconnected by six extracellular loops, four intracellular loops, and a large central cytoplasmic loop (CCL) or ICL3.^110^ Of these, the CCL has been identified as playing a critical role in maintaining protein function in *C. albicans*.^114^ In *C. parapsilosis*, although the function of *CpMDR1B* is similar to that of *C. albicans MDR1*, the deletion of *CpMDR1* has little to no effect on fluconazole susceptibility.^115^ At the same time, many studies have been conducted to determine which efflux pump plays a greater role in drug resistance in *C. auris*. In triazole-resistant isolates of *C. auris*, both *CDR1* and *MDR1* are more highly expressed than *CDR2* and *MDR2*. The deletion of *CDR1* alone in these isolates can significantly abrogate triazole resistance, whereas the deletion of *MDR1* does not affect the triazole MICs.^116^ This suggests that *CDR1* is more important than *MDR1* and that *MDR1* may work in conjunction with other factors in *C. auris*.

The expression of ABC efflux proteins in pathogenic *Candida* species is responsive to the activation of transcription factors that control their expression. Similarly, the expression of MFS proteins associated with multiple drug resistance can also be regulated by various transcription factors, including Cph1p, Mrr1p, Upc2p and Mcm1p.^110,117^ These factors regulate the expression of *MDR1* by binding to the promoter region of *MDR1*. Mrr1p, Upc2p and Mcm1p are identified as positive regulators, whereas Cph1p function as a negative regulator. Among these transcription factors, Mrr1p is the most important, and the transition to homozygous expression of CaMrr1p, which will lead to gain-of-function mutations leading to enhanced expression of the *MDR1* has been identified as a direct cause of *CaMDR1*-mediated azole resistance.^108^ Similar to *C. albicans*, *MRR1* mutations have also been confirmed to be associated with azole resistance in *C. parapsilosis*.^115^ The mutations in Mrr1p including I283R, R479K, A854V, G583R and K873N can lead to increased expression of *CpMDR1*. While an N647T amino acid substitution in Mrr1p has been identified in most fluconazole-resistant *C. auris* clade III isolates, the role of the transcription factor Mrr1p in azole resistance remains unclear.^118,119^ Additionally, this mutation consistently occurs alongside hotspot mutations in *ERG11*, which increases the ability of azole resistance.^118^

### Alteration in drug target

Mutations in *ERG11* can result in structural changes to the Erg11 protein, Erg11p, which can reduce the binding affinity of azole antifungals and, consequently, their effectiveness. Among *Candida* fungal strains, *ERG11* mutations related to antifungal resistance in *C. albicans* have been the most comprehensively studied.^120^ Over 140 amino acid substitutions in Erg11p have been reported in clinical isolates of *C. albicans*, with most of these substitutions occurring in three ‘hot spot’ regions of the protein: amino acids 105–165, 266–287, and 405–488.^120,121^ Evidence indicates that different amino acid substitutions contribute varying levels of antifungal resistance, and double substitutions are more effective than single substitutions in conferring drug resistance.^122^ Among all mutations, Y132F and K143R substitutions have been shown to play the most significant roles in fluconazole resistance in *C. albicans*. These mutations decrease azole binding affinity or interfere with substrate entry, thereby allowing the fungus to continue producing ergosterol even in the presence of the drug.^123,124^ Additionally, the K143R substitution has been reported to increase the expression of Erg11p and elevate ergosterol levels.^124^

The mechanism of *ERG11* mutation in non-albicans strains is not as well understood, but some substitutions have been reported. Mutations at positions Y141 and S410 are associated with azole resistance in *N. glabrata*.^125^ A single amino acid substitution, Y132F, encoded by the mutation *A395T*, was observed in fluconazole-resistant isolates of *C. tropicalis*.^126^ The substitution has been reported to alter the environment of heme by removing a hydrogen bond between heme and tyrosine. This change inhibits the interaction between azole nitrogen and the iron in the heme of Erg11p but retains the protein's ability to bind and metabolize the substrate to produce ergosterol.^127^ The Y132F substitution has also been noted in resistant isolates of *C. parapsilosis* and *C. auris*.^128,129^ Another *ERG11* mutation G458S, K134R and K128N have been observed in certain clusters in fluconazole-resistant *C. parapsilosis* isolates, but Y132F remains the dominant change globally.^130^

For the most common fluconazole-resistant isolate among *Candida* strains, *C. auris*, the peptide sequence of Erg11p is highly similar to that in *C. albicans*, which leads to similar mutations.^131^ Nearly all fluconazole-resistant *C. auris* clinical isolates exhibit three types of mutations in the gene: F126L (also known as VF125AL), Y132F and K143R. These mutations are clade-specific: F126L is uniquely found in clinical isolates from South Africa, while Y132F and K143R are predominantly found in South Asian and South American isolates.^21,131^ Although these three mutant *ERG11* alleles can increase fluconazole and voriconazole MICs by 8- to 16-fold, they seem to affect only the efficacy of these two azole antifungals and not impact the MIC for other triazoles^131^ Additionally, a new mutation, F444L, has been reported as a factor influencing azole resistance in clade IV (South American) clinical isolates of *C. auris*. F444L is located in a heme-binding region and may affect the affinity of azole antifungals.^132^

Interestingly, in comparison with fluconazole and voriconazole, the Erg11p substitutions have a lesser effect on the MICs of itraconazole and posaconazole.^122,131^ The lipophilic side chains of these two triazoles are thought to interact with additional residues along the enzyme's ligand access channel, which may contribute to the maintained activity.^131^

### Increased drug target

Overexpression of the *ERG11* gene leads to a higher concentration of the azole target, Erg11p, which can result in reduced susceptibility to azole antifungals by ensuring continued ergosterol synthesis (Table 3).^71,133^ Although *ERG11* overexpression is considered one of the molecular mechanisms of azole resistance, it is often reported in combination with other mutations rather than being the sole cause of drug resistance and has rarely been reported in non-albicans drug-resistant species.^43,134,135^

Ergosterol depletion by azole antifungals or other inhibitors is a common factor leading to this upregulation, which is often driven by gain-of-function (GOF) mutations in transcriptional regulators that control ergosterol biosynthesis.^43^ A prime example is Upc2p, a zinc-cluster transcription factor, which has been found to contribute to the overexpression of *ERG11*, particularly in *C. albicans* and *C. tropicalis*.^71,136^ Upc2p contains an amino-terminal DNA-binding domain and a carboxyl-terminal conserved domain. The C-terminal domain has been shown to recognize cellular ergosterol.^137^ When the C-terminal domain of Upc2p binds to ergosterol in a sterol-rich environment, Upc2p remains in the cytosol in a repressed form. However, upon ergosterol depletion by azole antifungal treatment, ligand-free Upc2p becomes active and translocates to the nucleus to activate transcription.^137^ GOF mutations in the C-terminal domain, such as G648D and A643V in *C. albicans* Upc2p, result in the activation of transcription factors that upregulate ergosterol synthesis.^138,139^ However, not all isolates with *ERG11* overexpression contain mutations in *UPC2*, indicating that other mechanisms may also contribute to *ERG11* overexpression.^136,140^

As previously mentioned, aneuploidy, the presence of one or more extra copy of the left arm of chromosome 5 can also increase drug target expression where additional copies of the *ERG11* gene on the isochromosome (i5(L)) increases levels of *ERG11* expression.^103^ This form of aneuploidy can mediate high-level resistance to fluconazole and other azoles. This can occur in the diploid *C. albicans* and there are suggestions that an extra-chromosomal copy of the *ERG11* gene can also occur as a result of experimental evolution in *C. auris* increasing the levels of resistance to fluconazole.^95,96,141^

### Drug influx

Changes in the uptake of drugs is well described as a mechanism affecting the susceptibility of bacteria to antimicrobial agents, but this is not well characterized in *Candida* spp. Studies have suggested that changes in the phospholipid content of the *C. albicans* membrane may affect azole accumulation (suggesting passive diffusion as the main uptake mechanism).^142^ This challenges previous studies, which had suggested the presence of a permeability barrier in resistant cells.^143^

### Bypassing Erg11p-mediated sterol biosynthesis

Azole antifungals can bind to Erg11p to inhibit the conversion of lanosterol to ergosterol. Normally, sterol C-5 desaturase, encoded by *ERG3*, catalyzes the conversion of lanosterol to a toxic sterol, 14α-methylergosta-8,24-dien-3,6-diol, which accumulates due to Erg11p inhibition by azoles.^127,144^ When a mutation in *ERG3* causes a defect in sterol C-5 desaturase, the accumulation of alternative, non-toxic 14α-methylsterol in the cell membrane can lead to azole cross-resistance.^144,145^ Some studies suggest that strains lacking ergosterol biosynthetic enzymes may activate stress-signalling cascades in response to antifungals.^144^ Furthermore, morphological regulators Efg1p is reported to participate in negatively regulating the expression of *ERG3*, and mutations in *EFG1* can increase susceptibility to antifungal agents in *C. albicans*.^146,147^

### Other mechanisms

#### Compensatory Stress Responses

In addition to resistance mechanisms that block drug binding to targets or reduce drug concentrations within the cell, *Candida* species have evolved cellular stress responses that provide protection from environmental stress, including antifungals. One of the key stress response components involved in azole resistance is Hsp90.

Hsp90 is a highly conserved molecular chaperone responsible for regulating the form and function of a wide range of substrate proteins.^148^ It preferentially interacts with a particular subset of the proteome, including key regulatory proteins of cellular signalling, making it more selective than other general chaperones.^149^

Hsp90 has been implicated in the potentiation of both basal tolerance to azoles and azole resistance emergence in multiple fungal species, including *C. albicans*.^150^ Two main mechanisms associated with azole resistance have been reported. Firstly, genetic depletion of Hsp90 has been linked to reduced decreased growth and maturation of *C. albicans* biofilms, which lead to reduced resistance of biofilms to commonly used azole antifungals *in vitro*.^151^ Secondly, Hsp90 stabilizes and maintains the function of several client proteins critical for fungal growth and survival under stress conditions, including calcineurin, Mkc1 and Cek1 MAP kinases.^148,152,153^ For example, mutations in Erg3p, which prevent the accumulation of toxic intermediates, are calcineurin-dependent.^148^ The regulation of client proteins by Hsp90 has been considered to play a broader role in drug resistance than Hsp90 alone.^148,152^ The importance of Hsp90 for enabling azole tolerance in *C. auris* has also been suggested.^148,154^

#### Biofilms

Since the demonstration of biofilm-mediated resistance in *C. albicans* in 1995, research on the role of biofilms in drug resistance in *Candida* species has gained increasing attention.^155,156^ Biofilms are described as communities of microorganisms that adhere to surfaces and are embedded in a self-produced matrix of extracellular polymeric substances and are recognized as important virulence factors.^157^ They can limit the penetration of substances through the matrix, protect fungal cells from host immune responses and can confer significant resistance to antifungals, as reviewed previously.^158^

*Candida albicans* is a highly pathogenic organism known for its ability to produce extensive biofilm structures.^159^ Cells of *C. albicans* within a biofilm have been shown to be more resistant to the antifungal fluconazole than planktonic cells. All non-*albicans Candida* species can form biofilms, but the extent and characteristics vary depending on the species/strains. For example, *N. glabrata* strains are less capable of forming biofilms compared to *C. parapsilosis* and *C. tropicalis*.^160^ The biofilm matrices of *C. parapsilosis* are thinner and contain large amounts of carbohydrates with less protein, whereas *C. tropicalis* biofilms consist of a dense network of yeast cells with low amounts of carbohydrates and proteins. In contrast, *N. glabrata* biofilms are more cohesive, with higher levels of both protein and carbohydrate.^155,160^

*Candida auris* represents a significant clinical challenge in controlling nosocomial infections. It exhibits similar virulence to *C. albicans* and *C. tropicalis* in *G. mellonella* models but does not show significant hyphae formation in larvae.^161^  *Candida auris* isolates can form large aggregates of cells both *in vitro* and *in vivo* due to incomplete budding, which provides resistance to antifungal agents in tissues and the environment.^161–163^ A recent study of 351 *C. auris* clinical strains suggests that fluconazole-susceptible isolates may be stronger biofilm formers than resistant isolates, perhaps associated with higher levels of oxidative stress tolerance, suggesting a more complex trade off that underlies biofilm-mediated azole resistance phenotypes.^164^ Fluconazole may also suppress the production of certain metabolites that promote biofilm formation in *C. auris*, further complicating the analysis.^165^ Further studies designed to unpick the molecular mechanisms of biofilm-mediated azole resistance, to develop better adjuvant approaches, are merited.

### Heteroresistance

Heteroresistance is the phenomenon where a clonal microbial population contains a small subpopulation of cells with significantly higher drug tolerance or resistance than the majority.^166,167^ In *Candida* species, even in isolates classified as azole-susceptible, rare cells (probably < 1%) can survive and grow at azole concentrations above the normal MIC of the strain.^168^ The phenotype of these heteroresistant subpopulations are usually unstable as they may arise under drug selection pressure but populations derived from such cells generally have a similar susceptibility to the original parental strain as the phenotype disappears or reverses once the drug is removed.^169–171^ Clinically, heteroresistance is a serious concern because the resistant subpopulation can expand during azole therapy, leading to treatment failure despite initial susceptibility testing indicating an azole-sensitive infection.^169,171^

Heteroresistance in fungi is influenced by dynamic molecular interactions at the genetic, transcriptional, and physiological levels.^166^ In *C. albicans*, this phenomenon is closely associated with the formation of aneuploid cells, which commonly involves duplication of chromosome 5.^172^ As previously mentioned, acquiring an extra copy of chromosome 5 can increase *ERG11* expression and also upregulate ABC transporter genes expression via *TAC1*, thereby simultaneously increasing the drug target abundance and the extent to which drugs are pumped drugs out of the cell. Additionally, stress-induced morphological forms such as trimeras promote chromosomal diversity and may facilitate the emergence of heteroresistant clones.^173^  *Nakaseomyces glabrata* exhibits similar strategies including the upregulation of efflux pumps.^174^ Interestingly, *N. glabrata* subpopulations can rapidly evolve into stable resistant mutants, which can easily blur the line between heteroresistance and stable azole resistance.^175^  *Candida parapsilosis* also shows potential for heteroresistance by forming trimeras and transient karyotypic changes observed under azole stress, although more commonly this species develops stable resistance.^173,176^

Across species, efflux pump overexpression, chromosomal rearrangements, and altered ergosterol biosynthesis collectively contribute to a reversible phenotype of a small, drug-tolerant subpopulation which can escape azole inhibition and may drive treatment failure if undetected. Clinically, the awareness of heteroresistance is prompting a re-evaluation of how we interpret antifungal susceptibility. Standard MIC tests may need to be supplemented with additional methods such as population analysis profiles or scrutiny of disk diffusion data to uncover heteroresistant subclones.^169^

## Future perspectives

The evaluation of azole resistance in *Candida* species has provided valuable insights into the mechanisms involved. Mutations in Erg11p have been consistently identified as a predominant form of azole resistance. Research at the genetic level remains a hot topic due to the involvement of multiple genes in azole resistance, although specific mutations in three ‘hot spot’ regions within *ERG11* have been identified.^120,121^ Currently, significant efforts are devoted to understanding the roles of efflux pumps in azole resistance, with a focus on the large binding pocket composed of critical amino acids that contribute to drug efflux. Understanding the importance of these amino acids in azole efflux could lead to the development of novel inhibitors targeting these critical amino acids.

Understanding azole resistance mechanisms leads to research focusing on overcoming this resistance. The most direct strategies for addressing azole resistance include designing new drugs and/or developing novel type of carrier system. Solid lipid nanoparticles (SLNs) and lipid core nanoparticles (LCNs), as novel carrier systems, have been developed to overcome azole resistance, particularly fluconazole resistance, by enhancing azole penetration and targeting, as well as maintaining azole concentration despite the function of efflux pumps.^177,178^ In the field of new drug development, on the one hand, modifications to the azole family, such as replacing triazole with tetrazole rings, have led to the development of new generations of azole antifungals, including VT-1161 (oteseconazole), VT-1129 (quilseconazole), and VT-1598, which are designed to evade target-based resistance mechanisms that affect older azoles.^179–181^ On the other hand, non-azole antifungals have been developed that show efficacy against *Candida* species, including azole-resistant strains, such as oral ibrexafungerp, a triterpenoid antifungal similar to echinocandins, and rezafungin, a newer echinocandin.^182,183^ Although developing new drug backbones requires extensive research and experimentation, these efforts offer promising directions for new drug development.

Drug combinations represent another strategy to address resistance. Combining azoles with other drugs can enhance efficacy and reduce the incidence of resistance, and this approach is widely used in treating various diseases.^184–186^ Contradictory results from different antifungal combinations, high costs, and serious side effects limit their use. Consequently, research has shifted towards combining antifungals with non-antifungal agents to overcome resistance. For instance, calcineurin disruption and calcium modulation play important roles in overcoming fluconazole resistance.^187,188^ Synergistic effects with azole antifungals have been observed with minocycline (a broad-spectrum antibiotic), tacrolimus (a calcineurin inhibitor), amiodarone (an anti-arrhythmic drug), alkaloids from *Tabernaemontana*, and Pseudolaric acid B from *Pseudolarix kaempferi*.^188–194^ Furthermore, combinations of efflux pump inhibitors (EPIs) have been considered as potential solutions to antibiotic resistance in recent years.^195^ Combining EPIs with antifungals should be an active area of research for overcoming azole resistance in *Candida* species, although there are currently few reports of synergistic effects of EPIs with azole antifungals.^196^ The use of AI and machine learning (AI-ML) models in predicting various drug combinations can significantly enhance the selection of optimal combinations to overcome specific resistance. These models can analyze vast datasets to identify synergistic effects between different drugs, improving the efficacy of antifungal treatments.

For patients at risk of *Candida* infections, vaccination could also be an effective solution to reduce the frequency of infections. Studies on anti-*Candida* vaccines focusing on diseases caused by *C. albicans*, including PEV7 and NDV-3, have been conducted.^197–199^ These vaccines target virulence factors and typically include live attenuated strains with impaired yeast-hyphae conversion or recombinant proteins from surface-located adhesion proteins. Challenges such as morphological, phenotypic and genetic variability must be overcome in vaccine development. Additionally, considering *Candida* species as common human commensals raises questions about whether vaccines might disrupt the balance of normal microbiota if they are not highly specific for pathogenic *Candida* species.

To mitigate the risks of *Candida* resistance to human health, it is crucial to further investigate unclear resistance mechanisms and focus on developing effective therapeutic methods. Understanding resistance at the genetic and amino acid levels will facilitate the design of novel azole antifungals with reduced resistance potential. Selective targeting of antifungals and vaccines is essential to improving efficacy and minimizing the transmission of *Candida* diseases.

## Conclusions

There has been a rise in invasive *Candida* fungal infections due to an increase in conditions leading to immune dysfunction. Meanwhile, the lack of appropriate treatment options due to fungal drug resistance has made treating deep-seated fungal diseases more challenging. Major mechanisms of azole resistance include alterations in *ERG11* and the overexpression of efflux pumps, with biofilms and Hsp90 also gaining attention. To address these issues, various strategies have been explored including combination therapies, developing new synthetic antifungals, such as fourth-generation azoles, and identifying new antifungals classes by exploring natural sources. The development of novel carrier systems and vaccines also offers promising new approaches to overcoming azole antifungal resistance. Continued research to identify new mechanisms, along with developing interventions, is essential to reduce the spread of azole resistance and preserve the efficacy of this important class of antifungal agents.
